# Equilibrium adsorption of polyvinylpyrrolidone and its role on thermoregulating microcapsules synthesis process

**DOI:** 10.1007/s00396-017-4061-5

**Published:** 2017-03-11

**Authors:** Anna M. Szczotok, Manuel Carmona, Anna-Lena Kjøniksen, Juan F. Rodriguez

**Affiliations:** 1 Department of Chemical Engineering, Institute of Chemical and Environmental Technology, University of Castilla-La Mancha, Avda. De Camilo Jose Cela s/n, 13071 Ciudad Real, Spain; 2 Faculty of Engineering, Østfold University College, P.O. Box 700, 1757 Halden, Norway

**Keywords:** Adsorption, Langmuir model, Microencapsulation, Phase change material, Suspending agent, Yield

## Abstract

The adsorption of polyvinylpyrrolidone (PVP) by the thermoregulating microcapsules has been studied. The mass ratio of PVP has been changed from 1 to 20, with respect to the lowest amount of PVP value (4.08 g). The results confirmed that a large amount of PVP was adsorbed by the polymeric shell. Experimental data were perfectly fitted by Langmuir model, obtaining at a confidence level of 95% values of 192.9 ± 0.4 g/kg and 0.18 ± 0.11 m^3^/kg for the maximum adsorption capacity and the equilibrium constant, respectively. It was found that utilizing PVP, at a concentration of 5.03 wt% of the total mass provided optimum conditions for synthesizing thermoregulating microcapsules containing Rubitherm®RT27 from poly(styrene-divinylbenzene) (P(St-DVB)), with the best thermal and physical properties. Finally, the robustness of the process was checked at a large scale by using a reactor that maintains geometrical similarities with that used at laboratory scale. The thermal properties, the encapsulation efficiency, and the microcapsule yield were similar, but at pilot plant scale, narrower particle size distributions were obtained.

## Introduction

The tensioactive agent is a key player in the processes of emulsion, suspension, particle, and capsule formation at nano- and microscale. Its influence and role on the particles size distribution, particle shape, agglomeration, or settling have been thoroughly studied [1–5]. Most of these studies pay all the attention on the ability of such compounds to modify the interfacial relations in terms of hydrophobicity, amphoteric properties, viscosity, and so on. Nevertheless, very few of such studies have considered not only the tensioactive as a skin of the particle but a constitutive portion of the material formed (particles or capsules).

Phase change materials (PCMs) are functional materials that can absorb energy (heat) during the melting process while keeping the temperature constant until the phase transition is completed. Moreover, during the solidification process, this energy will be released once again. Many scientists and companies have studied the use of PCMs in passive and active applications at low and middle temperatures. Among the passive storage systems in building applications, it is possible to distinguish PCM trombe walls, PCM wallboards, PCM shutter, PCM building blocks, air-based heating system, and ceiling boards. On the other hand, active storage systems include floor heating and ceiling boards [6]. Moreover, Kaygusuz and Ayhan [7] developed a solar heat pump system containing energy storage by encapsulated PCM for residential heating. They concluded that the highest saved net energy during the heating season was a dual source system (saving 12,056 kW), followed by a solar-assisted series of heat pumps (saving 10,120 kW) and a solar-assisted parallel system (saving 9390 kW). Further applications of PCM are ventilation nighttime cooling system as an alternative to air conditioning [8], thermoelectric refrigeration [9], and solar space heating [6], which are able to reduce energy consumption between 18 and 32%.

Different microencapsulation processes have been developed in order to solve the problems related with the PCM applications, such as leakage of the melted PCM, reactivity with the environment, volume change during the phase change transition state, and the heat transfer efficiency [10, 11]. Microencapsulation is a process where a thin shell is created around a microscopic droplet of active substance to produce capsules with useful properties. Solids, liquids, and gases can be encapsulated, and the size of microcapsules can range between 1 and 1000 μm, depending on the microencapsulation method. These methods can be categorized into chemical and physical processes. The most important techniques are interfacial polymerization [12–14], emulsion polymerization [15, 16], in situ polymerization [12, 17, 18], spray drying [5, 19], and suspension polymerization [20–22]. The selection of the encapsulation method is limited by the price of processing and core/shell requirement [23].

A large number of publications are related to the synthesis of microcapsules containing PCMs [4, 21, 22, 24–26]. However, only few of them have examined how the stabilizers affect the properties of the product and the microcapsule yield. The adsorption of nonionic surfactants onto solid surfaces has been studied previously [27–30], observing that a large amount of the suspending agent is loaded onto the solid material. Smith et al. [30] found a high-affinity adsorption of PVP onto polystyrene lattices in water, where the maximum adsorption capacity was independent on the PVP molecular weight. On the contrary, when they used lattices in 0.5 N of NaCl, the adsorption of PVP onto polystyrene lattices increased with the molecular weight. In the same way, Geffroy et al. [29] studied the adsorption of different nonionic surfactants formed by combining alkyl groups C_8_ or C_12_ with an ethylene oxide oligomer (EO). They found that the maximum adsorption capacity varied with the size of the polar headgroup. Hence, it is possible that surfactant agents could be incorporated to the microcapsules containing thermoregulating materials, and the quantity may depend on the molecular weight and polarity of the surfactants. Thus, a part of its tensioactive function, this compound is incorporated as a constitutive part of the microcapsule shell. This incorporation will increase the apparent microcapsule yield. However, there is a general lack of knowledge on the way in which the inclusion of the tensioactive agents influences not only the size/shape but also the intrinsic properties of the microparticles or microcapsules.

In previous studies, it was found that PVP is a suitable suspending agent for the production of microcapsules from styrene (St) and methyl methacrylate-styrene (MMA-St) copolymer containing PCMs [4, 21, 22, 31]. Nevertheless, thermoregulating microcapsules from styrene and divinylbenzene (DVB) with a spherical shape, smooth surface, and a high mechanical resistance, similar to those of ion exchange resins, have not been reported in the literature. The greater physical resistance of the cross-linked shell of these materials is necessary for their employment in active thermo-accumulating systems in which the particles have to be circulated and pumped by ducts as slurries. An approach to these kinds of materials is reported by You et al. [26] and Li et al. [32] encapsulating n-octadecane. However, the microcapsules present holes, making them concave in shape. Alcazar et al. [33] obtained the desired physical and mechanical properties encapsulating extractant agents from poly(styrene-divinylbenzene) by using toluene and a mixture of arabic gum and poly(vinyl alcohol) (GA:PVA) as porogen and suspending agents, respectively. Hence, in the present study, the applicability of PVP and toluene for producing thermoregulating microcapsules from poly(styrene-divinylbenzene) containing Rubitherm®RT27 is examined. In addition, the microcapsule yield, the adsorption of PVP in the final product, and the robustness of the process at large scale have been studied.

## Materials and methods

### Materials

The monomers, styrene of reagent grade (St, 99 wt%, Sigma-Aldrich Chemical Co.), and divinylbenzene of technical grade (DVB, containing 80% DVB isomers, Sigma-Aldrich Chemical Co.) were purified by washing with an aqueous sodium hydroxide solution (1.25 N) and calcium chloride as desiccant. The remaining reagents were used as received, without further purification. Benzoyl peroxide (BPO, humidified with ∼25% of H_2_O pure, pharma grade, PanReac Co.) was used as initiator. Rubitherm®RT27 was used as core material. Polyvinylpyrrolidone (PVP, K30, M_w_ 40,000*g* mol^−1^, Sigma-Aldrich Chemical Co.) of reagent grade was used as suspending agent. Toluene and ethanol of reagent grade was used as inert diluents, and cleaner of the samples were supplied by Sigma-Aldrich Chemical Co. Water with a conductivity of 1 μS/cm was produced in our laboratory by distillation followed by deionization using ion exchange. Nitrogen was high-purity grade.

### Synthesis of microparticles

Microcapsules were prepared by a suspension-like polymerization technique based on the recipe used by Alcazar et al. [33]. Polymerization reactions were performed in a 0.5-L jacketed glass reactor equipped with a reflux condenser, a nitrogen gas inlet tube, a digital control of stirring, and a thermostatic bath to keep the reaction at the required conditions. The installation set up was described in detail in [21].

As mentioned above, the synthesis include two phases: a continuous phase containing water and the suspending agent, and a discontinuous phase containing styrene, divinylbenzene, Rubitherm®RT27, toluene, and benzoyl peroxide. The recipe for the microcapsule synthesis is shown in Table 1. In order to obtain the desired microparticles, the continuous phase was transferred to the glass reactor fixing the agitation at 800 rpm and the temperature at 80 °C. The initiator was dissolved and premixed with monomers and the Rubitherm®RT27 at 50 °C, allowing an efficient polymerization reaction and avoiding idle time. When the discontinuous phase was added into the continuous phase, the polymerization process was carried out for 5 h under a nitrogen atmosphere. After polymerization, the product was purified by repeated washing with ethanol and filtrated to remove impurities. Finally, the product was left dried at room temperature for at least 24 h.

**Table 1:** The initial recipe used for obtaining microcapsules with Rubitherm®RT27

	Ingredient	Weight
Continuous phase (g)	Water (Mili-Q)	350.00
	Polyvinylpyrrolidone (PVP)	4.08–81.60
Discontinuous phase (g)	Rubitherm®RT27	32.34
	Styrene (St)	7.45
	Divinylbenzene (DVB)	7.45
	Toluene	62.03
	Benzoyl peroxide (BPO)	3.37

**Fig. 1:**
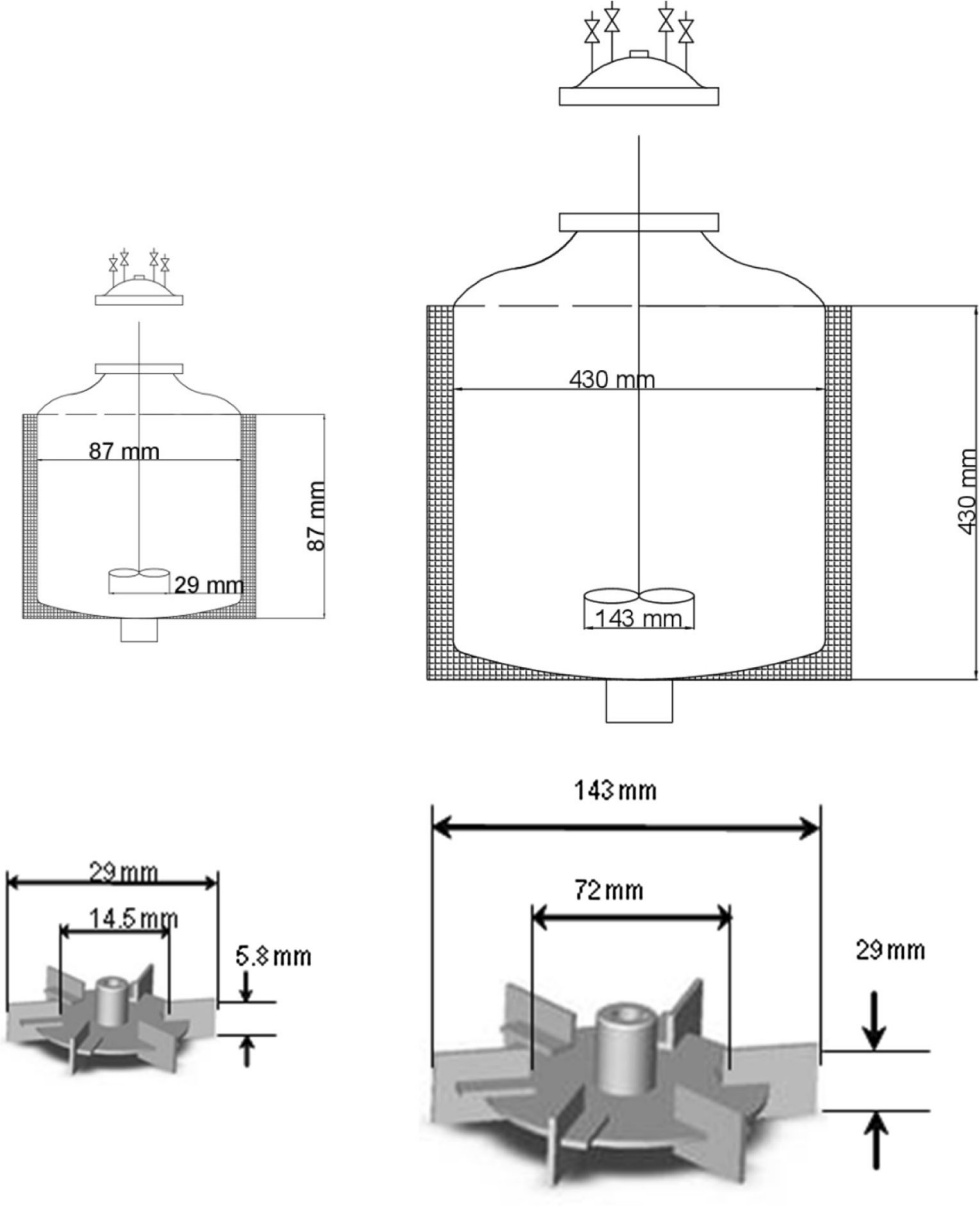
Scheme of the reactors and impeller dimensions for laboratory and pilot plant scales

Seven different experiments were performed changing the mass of PVP from 4.08 to 81.60 g, following the sequence PVP_1_, PVP_2_, PVP_4_, PVP_6_, PVP_8_, PVP_10_, and PVP_20_. PVP_i_ corresponds to the mass ratio between the specific experimental PVP compared to the lowest value used. Furthermore, three additional experiments were carried out in a 100-L reactor, maintaining the same temperature, using the following PVP mass ratios: PVP_2_, PVP_6_, and PVP_10_ but a decreasing agitation rate of 300 rpm. The design properties of both reactors are shown in Fig. 1. The 100-L reactor maintains geometrical similarities to the reactor used at lab scale. The dimensionless correlations in diameter of the reactor and Rushton impeller were established based on the stirrer configuration reported by Shäfer et al. [34],

1 $$ \frac{d_1}{D}=\frac{1}{3} $$

2 $$ \frac{H}{D}=1 $$

3 $$ \frac{W}{d_1}=\frac{1}{5} $$

4 $$ \frac{L}{d_1}=\frac{1}{4} $$

5 $$ \frac{d_2}{d_1}=\frac{3}{4} $$

where *D* is the reactor diameter (m), *H* is the height of the liquid (m), *d*_1_ is the impeller diameter (m), *d*_2_ is the disc diameter (m), and *W* and *L* are the weight and length of the blade (m), respectively. The repeatability and robustness of the microencapsulation process were confirmed performing every experiment three times.

### Characterization

#### Yield

The microcapsule yield (η_r_) was determined by considering the maximum amount of the product as that constituted by the polymer from monomers P(St-DVB)_MC_, and encapsulated Rubitherm®RT27 (RT27_MC_) by:

6 $$ {\upeta}_{\mathrm{r}}=\frac{\mathrm{RT}{27}_{\mathrm{MC}}+\mathrm{P}{\left(\mathrm{St}-\mathrm{DVB}\right)}_{\mathrm{MC}}}{\mathrm{RT}{27}_{\mathrm{feed}}+{\left(\mathrm{St}-\mathrm{DVB}\right)}_{\mathrm{feed}}} $$

where RT27_feed_ and (St-DVB)_feed_ are the weights of the Rubitherm®RT27 and monomers fed to the reactor, respectively.

#### Differential scanning calorimetry

Measurements of melting point and latent heat storage capacities of different materials were performed in a differential scanning calorimetry (DSC) model Q100 from TA Instruments, equipped with a refrigerated cooling system and nitrogen as the purge gas. Measurements were carried out in the temperature range from −40 to 80 °C with heating and cooling rate of 3 °C/min.

The paraffin content (C_PCM_) in the microcapsule was calculated based on the enthalpy values:

7 $$ {\mathrm{C}}_{\mathrm{PCM}}\ \left(\%\right)=\frac{{\varDelta \mathrm{H}}_{\mathrm{MC}}}{{\varDelta \mathrm{H}}_{\mathrm{RT}27}}\times 100\% $$

where ΔH_MC_ is the enthalpy for the analyzed microcapsules (J/g) and ΔH_RT27_ is the enthalpy of pure Rubitherm®RT27 (171.2 J/g). The encapsulation efficiency (EE) can be calculated from the relationship between the Rubitherm®RT27 inside the total microcapsules (RT27_MC_) and the Rubitherm®RT27 fed (RT27_feed_):

8 $$ \mathrm{EE}\ \left(\%\right)=\frac{{\mathrm{RT}27}_{\mathrm{MC}}}{{\mathrm{RT}27}_{\mathrm{feed}}}\times 100\% $$

Microcapsules can contain Rubitherm®RT27, monomers, toluene, and suspending agent. In order to know the contribution of these different compounds, thermal analyses were carried out.

#### Thermogravimetric analysis

The thermal stability, amount of Rubitherm®RT27, and toluene content of the synthesized microcapsules were obtained by using the TA instruments SDT Q600 Simultaneous DSC-TGA from room temperature to 600 °C at a heating rate of 10 °C/min under a nitrogen atmosphere.

#### Scanning electron microscopy

The morphology and the surface features of the microcapsules were observed by using Quanta 250 (FEI Company) with a tungsten filament operating at a working potential 12.5 or 15 kV equipped with an EDAX Apollo X (AMETEK), an energy dispersive x-ray spectrometer (EDX), which analyze the chemical composition of the samples with the detection limits about 1000 ppm or 0.1 wt%.

#### Particle size and particle size distribution

Volume average particle size (dv_0.5_) and number average particle size (dn_0.5_) of the microcapsules were determined by low-angle laser light scattering (LALLS) laser diffraction, utilizing a Malvern Mastersizer 2000 equipped with a Scirocco 2000 unit for analyzing dispersions of the particles in air and a software that uses the Mie theory to analyze the experimental data.

## Results and discussion

**Fig. 2:**
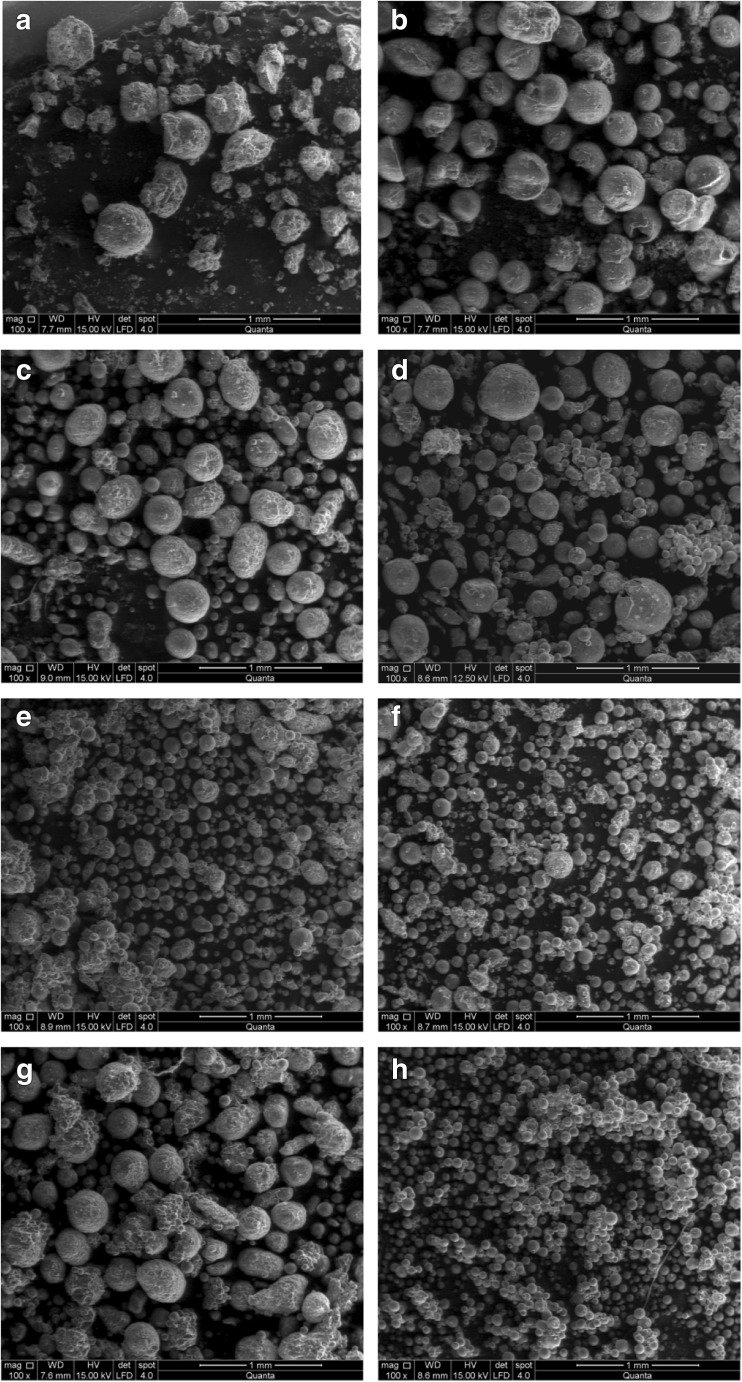
SEM micrographs of microcapsules synthesized by different amounts of stabilizers. **a** MC(PVP_1_). **b** MC(PVP_2_). **c** MC(PVP_4_). **d** MC(PVP_6_). **e** MC(PVP_8_). **f** MC(PVP_10_). **g** MC(PVP_20_). **h** MC(PVP_6_)_100L_

Figure 2 shows the effect of the amount of PVP on the morphology of the synthesized microcapsules at lab scale and also an example of the product obtained at pilot plant scale. The only product that presents an irregular shape was produced by using a mass ratio PVP_1_ (Fig. 2a). Accordingly, this amount of surfactant is not enough to ensure a good dispersion of the discontinuous phase into the continuous one. All the other products have a spherical shape and a smooth surface, also indicating that the use of toluene as porogen prevent the formation of holes.

**Fig. 3:**
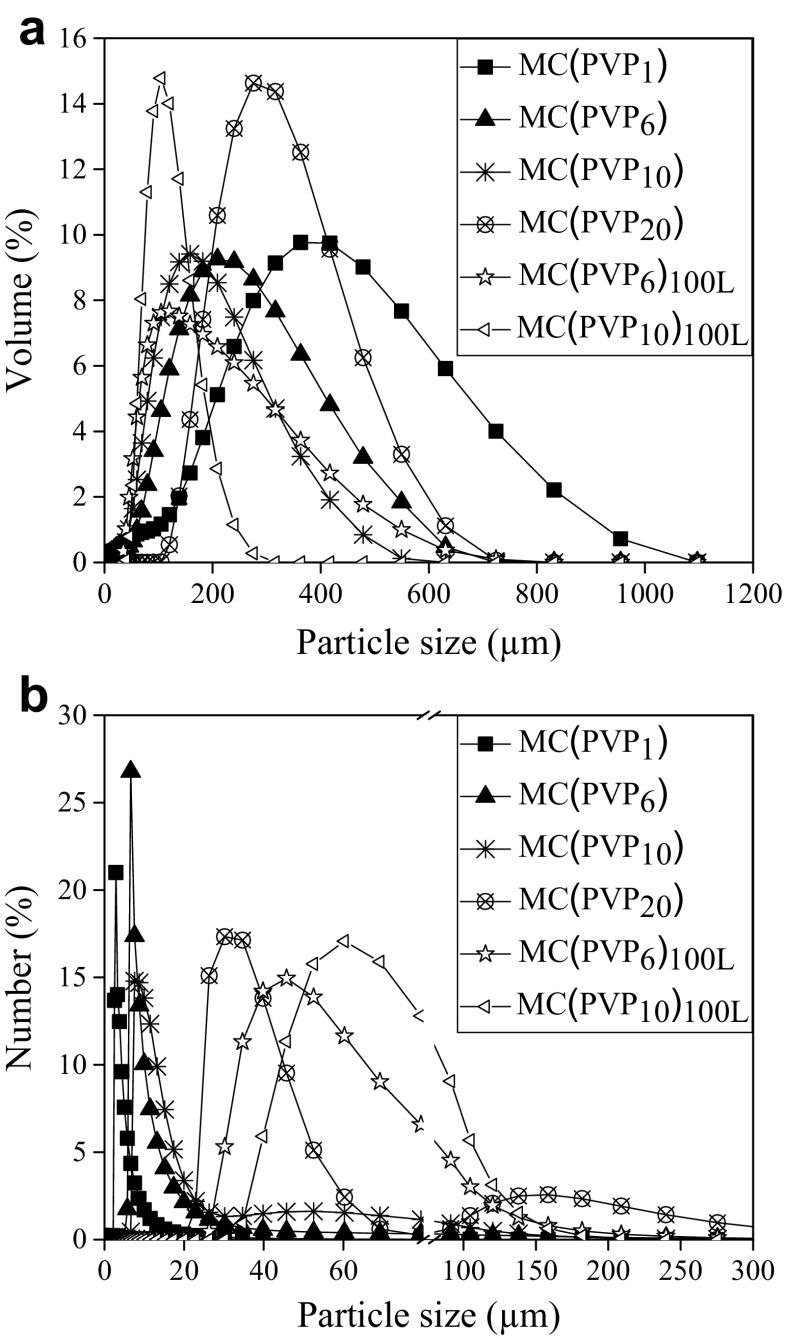
Particle size distribution for microcapsules with different amounts of PVP. **a** Volume average. **b** Number average

It is also observed that all products seem to be quite homogeneous in particle size. As can be seen from Fig. 3, the particle size is decreasing with the amount of PVP, except for the highest PVP concentration (PVP_20_). This indicates that a large amount of suspending agent is unfavorable for the formation of single particles, promoting the coagulation process.

An increase in the amount of stabilizer from a mass ratio PVP_1_ to PVP_10_ results in a product with the desired properties: smaller particle size, spherical and regular shape, and also a smooth surface. It was observed that the product having the smallest particle size in volume was achieved by using the reactor of 100 L and a mass ratio PVP_10_. This result could be related with the energy dissipation rate. If the energy delivered by the stirrer at both scales were the same, the particle size should be equal [31]. The particle size of the microcapsules is smaller in the pilot plant run even though a much slower stirring rate was used at pilot plant scale (300 rpm) than at laboratory scale (800 rpm). This means that the geometrically similar Rushton stirrer utilized in the pilot plant is more efficient in delivering energy to the bulk.

According to the SEM photographs, the optimal PVP mass ratio must be between PVP_6_ and PVP_10_. The results illustrate that it is possible to synthesize microcapsules (MC(PVP_i_)) by this proposed method, satisfying user’s requirements in lab scale as well as in pilot plant.

The volume and the number average particle size distribution of the different products have been analyzed by laser diffraction, and the results are shown in Fig. 3.

Figure 3a indicates that all products present unimodal volume average distributions. As expected, the widest distribution was observed for the irregular product obtained using a mass ratio PVP_1_. However, Fig. 3b shows that the number average particle size distribution for the mass ratio PVP_10_ and PVP_20_ at lab scale are bimodal. These results confirm that an excess of suspending agent favors the formation of smaller particles, which agglomerates during the reaction time, promoting the formation of larger particles. Hence, in order to obtain unimodal distributions, a mass ratio of PVP_6_ should be used at lab scale. Nevertheless, Fig. 3 also illustrates that the number and the volume average particle size distributions for the product obtained at pilot plant scale are very similar to each other at mass ratio PVP_10_, indicating that it is possible to obtain monodisperse materials in this conditions.

**Fig. 4:**
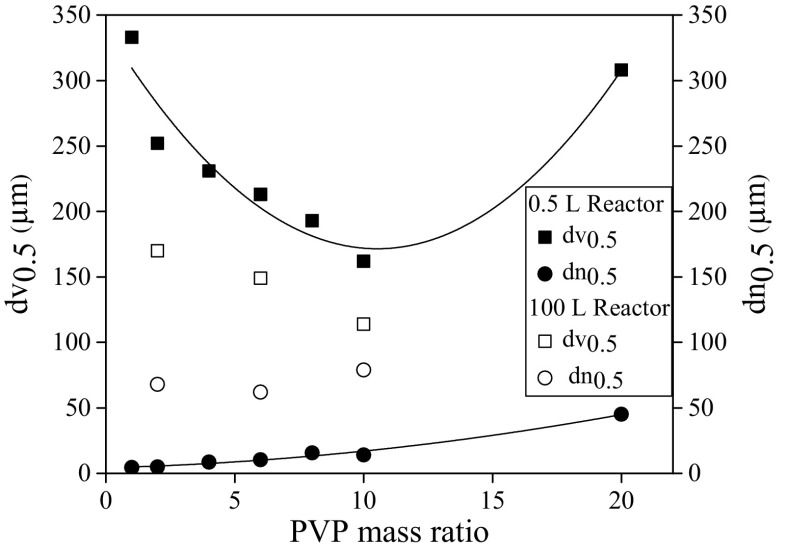
Influence of PVP concentration on the volume average (dv_0.5_) and number average (dn_0.5_) particle size

Figure 4 shows the influence of the amount of PVP on the volume average and the number average particle size.

As can be seen, the volume and number average sizes do not follow the same trend. While the behavior of the number average increases with the PVP mass ratio and seems to be nearly linear, the volume average presents a minimum at PVP_10.5_ at lab scale. The number average sizes are more sensitive to the presence of small particles, while the volume average sizes are biased toward large particles. Accordingly, the results suggest that as the concentration of PVP is increased up to PVP_10_, fewer of the largest particles are produced (reduction in dv_0.5_) combined with fewer very small particles (increase in dn_0.5_) at both studies. In general, increasing the mass ratio of PVP in pilot plant scale, all products present a larger particle size in number but a lower particle size in volume than those obtained at lab scale, being closer to the gap between them and indicating the possibility to synthesize monodisperse material.

The minimum in the volume average sizes and the increase in the number average sizes may be related to the solubility of PVP in water. According to the data sheet from the PVP supplier, at a molecular weight of 40,000 g/mol, the solubility is 100 mg/mL at 25 °C. This is lower than the 233 mg/mL of the PVP_20_. At the highest concentration, some of the PVP with therefore be in solid state, dispersed as small particles in the solvent. These solid particles may act as nuclei on which the polymer drops grow, leading to the formation larger microcapsules. In the case of the 100-L reactor, the volume and number averages are closer to each other than for the lab-scale experiments, which is indicative of a narrower size distribution. Two main conclusions can be extracted from these observations: (1) for devices that are geometrically similar, the power delivery is more effective at large scale. (2) When increasing the scale using the same mass ratio, it is possible to reach a more monodisperse product.

These results are not in agreement with those reported by Ma et al. [35], obtaining stable particle size from 21.6 to 20.9 μm when the PVP concentration was changed from 2.0 to 7.0 g in 225 g of water, respectively. In addition, they obtained an increase in the amount of coagulum for a PVP concentration higher than 4.0 g in 225 g of water, a phenomenon that only appears in our case for the largest studied PVP mass ratio (PVP_20_).

**Fig. 5:**
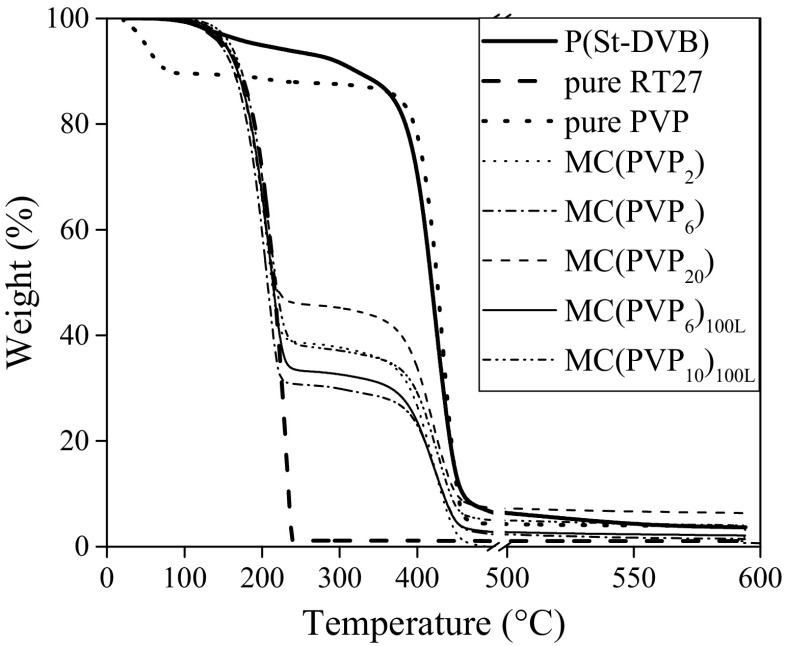
TGA curves for the studied materials: P(St-DVB) copolymer, pure Rubitherm®RT27, PVP, and microcapsules synthesized by using the different PVP mass ratios

In order to examine the presence of toluene and PVP in the microcapsules, thermal analyses were performed. The TGA curves for the pure Rubitherm®RT27, St-DVB copolymer, pure PVP, and some microcapsules obtained at different PVP mass ratios were shown in Fig. 5.

This figure shows that the pure material with the greatest volatility is the Rubitherm®RT27, followed by the evaporation of the Rubitherm®RT27 encapsulated by the polymeric shell and finally, the decomposition processes of P(St-DVB) and PVP. It is also observed that while the Rubitherm®RT27 presents only one weight loss peak from 150 to 250 °C, polymer materials (PVP and P(St-DVB)) and microcapsules exhibit two different weight losses. In the case of PVP, the first weight loss takes place at 100 °C, and it is related with the water content, whereas for P(St-DVB) and microcapsules, the first weight loss appears from 120 to 220 °C, due to the evaporation of monomer, Rubitherm®RT27, and polymer having a low molecular weight. Hence, MC(PVP_6_) are the microcapsules with the highest content of Rubitherm®RT27, followed by the MC(PVP_6_)_100L_, and obtaining a minimum in the case of MC(PVP)_20_. These results indicate that the larger the mass ratio of PVP, the lower the paraffin content in the microcapsules.

The second weight loss is due to the polymer degradation, being the mean degradation temperatures 431.87 and 422.07 °C for PVP and P(St-DVB), respectively. Therefore, the degradation temperatures of PVP and P(St-DVB) suggest that TGA is not a good technique to differ between the presence of PVP or P(St-DVB) in the microcapsules. Finally, the amount of residue obtained in this TGA after 500 °C shows that Rubitherm®RT27 is completely evaporated, but the polymeric materials, PVP and P(St-DVB), present practically the same amount of residue, indicating that the PVP could be present in the copolymer, since it was synthesized by using PVP as a surfactant agent. In the case of microcapsules, the residue increases with the mass ratio of PVP used in the recipe, explaining why the microcapsules produced by using a high mass ratio of PVP have a lower paraffin content.

**Fig. 6:**
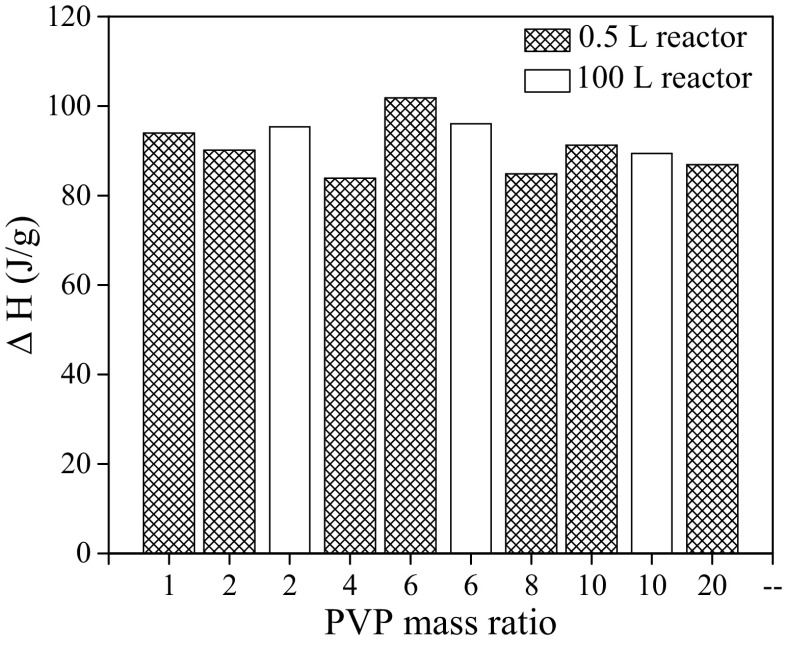
Latent heat of the microcapsules as function of the PVP mass ratio

Figure 6 shows latent heat (ΔH) of the different synthesized microcapsules. These results indicate that using this technology, microcapsules with thermal energy storage (TES) capacity higher than 80 J/g can be produced. The maximum values 101.80 and 96.08 J/g were reached for the microcapsules synthesized using a mass ratio of PVP_6_ at laboratory and pilot plant scales, respectively. This does not follow the observation of Li et al. [36] where a two-step miniemulsion polymerization method resulted in an increase in the latent heat of the microcapsules from 114.6 to 143.7 J/g when the amount of surfactant was changed from 0.05 to 0.20 g in the water. This indicates that thermal properties of microcapsules are mainly dependent on the microencapsulation technology.

**Fig. 7:**
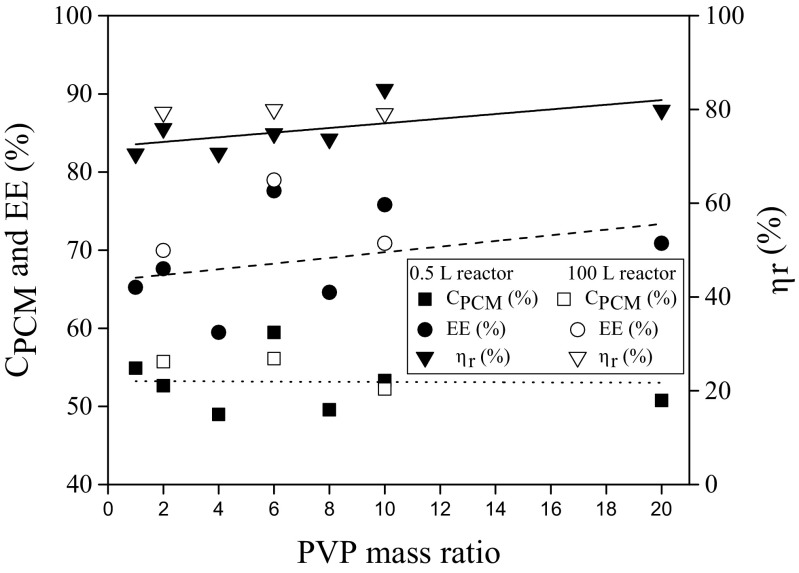
Effect of PVP amount on microcapsule yield (η_r_), paraffin content (C_PCM_), and encapsulation efficiency (EE)

According to the latent heat, PVP_6_ can be considered as the optimal PVP mass ratio to produce microcapsules having a large TES capacity. Nevertheless, before taking a final decision about the optimal recipe, the effect of PVP mass ratio on other important variables was studied. As it has been stated in the introduction, the final goal of the article is to clarify the role of the tensioactive agent not only on the particle size but also on other important variables such as the encapsulation efficiency, the paraffin content, and the microcapsule yield. The hypothesis is that the tensioactive agent remains as a constitutive part of the particle after the synthesis, with significant influence on its final properties. The paraffin content (C_PCM_), encapsulation efficiency (EE), and the microcapsule yield (η_r_) are depicted in Fig. 7 as function of the PVP mass ratio.

A slight increase of EE and η_r_ with the mass ratio of PVP is observed whereas the C_PCM_ seems to be almost stable at 53%, although a highest value of 59.46% was obtained for PVP_6_. These values of C_PCM_ are twice as high as that (28.69 wt%) reported by Sanchez et al. [21] synthesizing microcapsules containing Rubitherm®RT27 from polystyrene but in the absence of a porogen agent.

The highest values of encapsulation efficiency (77.59%) and microcapsule yield (84.28%) were found for the products using mass ratios of PVP_6_ and PVP_10_, respectively. These results are contrary to those reported by Khakzad et al. [25] encapsulating hexadecane in a melamine formaldehyde shell by using the in situ dispersion polymerization technique in aqueous media. They observed a decrease in the encapsulation efficiency from 121 to 84.4%, increasing the poly(vinyl alcohol) concentration from 1 to 8 wt%. In the case of the yield, Ma et al. [35] synthesizing polystyrene-polyacrylamide composite microspheres from water/oil/water emulsion and further suspension polymerization reported a maximum of 90.2% when they used 5 g of PVP in 225 g water. Hence, there is no rule that allows establishing the optimum concentration of the stabilizer in the manufacture of microcapsules, as it is dependent on the microencapsulation technology. As expected, values of C_PCM_, EE, and η_r_ obtained at pilot plant scale are similar to those reached at laboratory scale but requiring a lower amount of suspending agent for leading the same encapsulation efficiency and microcapsule yield (78.86 and 79.99%, respectively for mass ratio PVP_6_). Hence, by changing the PVP mass ratio, it is possible to increase the paraffin content and to obtain microcapsules with the desired characteristics.

As commented above, the suspending agent forms part of the microcapsules. The presence of PVP in the microcapsules (*f SA*) can be quantified from the DSC analyses and weighing the total amount of product (P_MC_) obtained in each reaction. A 100% of monomer conversion was assumed.

9 $$ {P}_{MC}={RT27}_{MC}+ P{\left( St- DVB\right)}_{MC}+ f\  SA $$

where *SA* is the total amount of the suspending agent used in the synthesis.

In this way, the fraction of suspending agent that constitutes the microcapsules *f* can be calculated by:

10 $$ f=\frac{P_{MC}- RT{27}_{MC}- P{\left( St- DVB\right)}_{MC}}{SA} $$

Once values of *f* are calculated, the amount of PVP on the microcapsules (Г in g/kg) and the concentration of PVP in the bulk solution at the end of the process (C_b_ in kg/m^3^) can be estimated by Eqs. 11 and 12, respectively.

11 $$ \varGamma =\frac{fSA}{P_{MC}} $$

12 $$ {C}_b=\frac{\left(1- f\right) SA}{V} $$

where *V* is the total volume of the bulk solution.

**Fig. 8:**
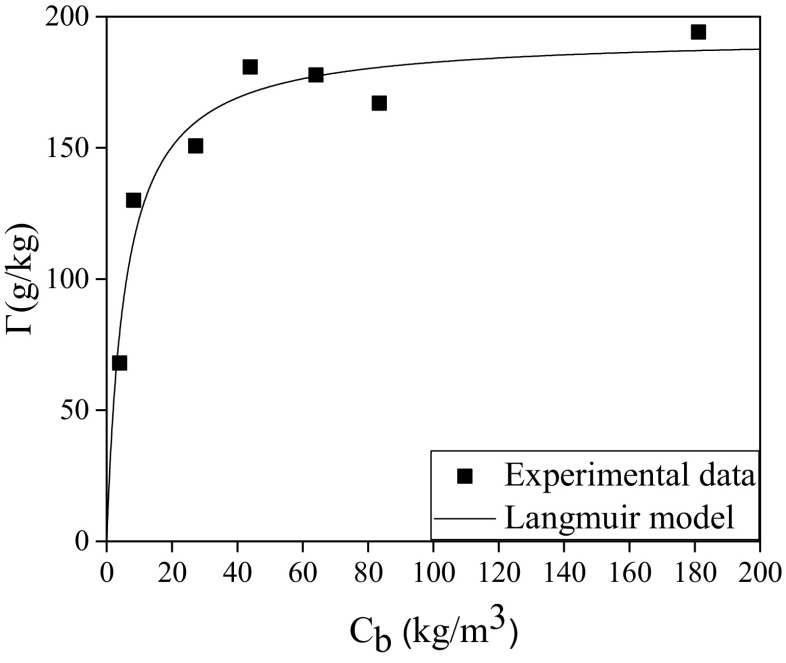
Adsorption isotherm of PVP onto P(St-DVB) in water/toluene media at 80 °C

Figure 8 shows the relationship between Г and C_b_. According to this figure, PVP is strongly loaded by P(St-DVB) even at a very low concentration in water/toluene media. This behavior is a characteristic of those sorbents that present a high affinity for the solute in adsorption systems [30]. Besides, as in most of the conventional adsorption process, it was observed that a maximum value of PVP incorporation to the microcapsules is reached. The interaction established between the P(St-DVB) and PVP can be explained by the observations of Smith et al. [30]. They studied the adsorption of PVP onto polystyrene lattice and concluded that “in water, interaction with the PSt occurred through the PVP hydrophobic methylene/methane groups and the positive dipole of the amide nitrogen of the pyrrolidone ring. The negative dipole associated with the amide oxygen is directed away from the surface into the solution”.

In order to fit these data, the Langmuir model (Eq. 13) usable for monolayer materials was selected [27].

13 $$ \frac{\varGamma}{\varGamma^{max}}=\frac{K{C}_b}{1+ K{C}_b} $$

where *Г* ^max^ is the maximum retention capacity of the P(St-DVB) and *K* is the equilibrium constant of the system, defined as the ratio between adsorption and desorption rate constants, respectively.

Experimental data were fitted to Eq. 13 in order to obtain the two unknown parameters *Г* ^max^ and *K*. For that purpose, a fitting tool for solving non-linear equations based on Marquardt’s algorithm was utilized. The fitting values of *Г* ^max^ and *K* and their confidence interval using a confidence level of 95% (*α* = 0.05) were 192.9 ± 0.4 g/kg and 0.18 ± 0.11 m^3^/kg, respectively. The proposed model gives a good fit to the experimental data, illustrating that the distribution of the PVP between the solid and liquid phases at the end of the polymerization process follows a Langmuir type trend.

The value of *Г* for MC(PVP_6_) was also confirmed by EDAX analyzing the nitrogen content of the microcapsules. In this way, EDAX analyses were performed in MC(PVP_6_) on the external surface and at the center of the microcapsule. A small difference was found between the external and internal nitrogen contents 1.46 and 2.30 wt%, respectively. These values are within the respective theoretical nitrogen content (2.10 wt%) obtained from the adsorption curve (167.01 g/kg) and the nitrogen content of the PVP (12.60 wt%). This distribution of nitrogen through the microcapsules also indicates that the adsorption of PVP takes place in the whole microcapsule structure.

Hence, thermoregulating microcapsules from P(St-DVB) could contain up to a 19.3 wt% of surfactant when the percentage of PVP of the total mass is higher than 5.03 wt%. Moreover, a large amount of PVP decreased the particle size of the microcapsules, and its presence in the final product was undesirable, as it prevents reaching a high TES capacity. Finally, regarding the physical and thermal properties, PVP_6_ is the most suitable PVP mass ratio, because it allows the production of microcapsules with the highest thermal energy storage capacity (101.8 J/g), while obtaining spherical particles with a uniform and small size distribution and the lowest formation of agglomerated material. Besides, the good results reached at pilot plant scale confirm the robustness of the selected conditions and the technology for manufacturing thermoregulating microcapsules.

## Conclusion

An important incorporation of the surfactant agent into the thermoregulating microcapsules was confirmed. It was found that PVP is retained by the P(St-DVB) microcapsules. The distribution of the PVP between the solid and liquid phases at the end of the polymerization process follows a Langmuir type trend. The PVP distribution data were fitted by the Langmuir model obtaining a value of 192.9 g/kg and 0.18 m^3^/kg for the maximum retention capacity and the equilibrium constant, respectively. The adsorption of surfactant agents on polymeric materials has been reported previously by other authors [28, 29], but as far as we know not quantified and modeled for thermoregulating microcapsules. In this way, kinetic studies for determining the uptake of PVP by microcapsules from P(St-DVB) as function on the time must be accomplished in the future [27]. The amount of surfactant is shown to be a key variable in order to synthesize thermoregulating microcapsules with the desired characteristics. By using a mass ratio of PVP_6_ (5.03 wt%), microcapsules containing Rubitherm®RT27 with the best thermal and physical properties were obtained. Finally, the robustness of the process was checked at pilot plant scale, obtaining more monodisperse materials, a TES capacity of 96.1 J/g, an encapsulation efficiency of 79.0% and a microcapsule yield of 80.0%. These characteristics were similar to those obtained at laboratory scale, although the particle size distribution was improved at pilot plant scale.
